# Effects of hypoxia on the proliferation, mineralization and ultrastructure of human periodontal ligament fibroblasts *in vitro*

**DOI:** 10.3892/etm.2013.1349

**Published:** 2013-10-16

**Authors:** HAI-YUAN ZHANG, RUI LIU, YONG-JUN XING, PING XU, YAN LI, CHEN-JUN LI

**Affiliations:** 1 Department of Stomatology, Chengdu Military General Hospital of PLA, Chengdu, Sichuan 610083, P.R. China; 2 Department of Stomatology, Research Institute of Surgery and Daping Hospital, The Third Military Medical University, Chongqing 400042, P.R. China

**Keywords:** periodontal disease, hypoxia, human periodontal ligament fibroblasts, proliferation, mineralization, ultrastructure

## Abstract

This study aimed to investigate the effects of hypoxia on the proliferation, mineralization and ultrastructure of human periodontal ligament fibroblasts (HPLFs) at various times *in vitro* in order to further study plateau-hypoxia-induced periodontal disease. HPLFs (fifth passage) cultured by the tissue culture method were assigned to the slight (5% O_2_), middle (2% O_2_), and severe hypoxia (1% O_2_) groups and the control (21% O_2_) group, respectively. At 12, 24, 48 and 72 h, the proliferation and alkaline phosphatase (ALP) activities were detected. The ultrastructure of the severe hypoxia group was observed. HPLFs grew more rapidly with an increase in the degree of hypoxia at 12 and 24 h, and significant levels of proliferation (P<0.05) were observed in the severe hypoxia group at 24 h. Cell growth was restrained with an increase in the degree of hypoxia at 48 and 72 h, and the restrictions were clear (P<0.05) in the middle and severe hypoxia groups. ALP activity was restrained with increasing hypoxia at each time point. The restrictions were marked (P<0.05) in the severe hypoxia group at 24 h and in the middle and severe hypoxia groups at 48 and 72 h. However, the restriction was more marked (P<0.05) in the severe hypoxia group at 72 h. An increase was observed in the number of mitochondria and rough endoplasmic reticula (RER), with slightly expanded but complete membrane structures, in the severe hypoxia group at 24 h. At 48 h, the number of mitochondria and RER decreased as the mitochondria increased in size. Furthermore, mitochondrial cristae appeared to be vague, and a RER structural disorder was observed. At 72 h, the number of mitochondria and RER decreased further when the mitochondrial cristae were broken, vacuolar degeneration occurred, and the RER particles were reduced while the number of lysosomes increased. HPLF proliferation and mineralization was restrained. Additionally, HPLF structure was broken for a relatively long period of time in the middle and severe hypoxia groups. This finding demonstrated that hypoxia was capable of damaging the metabolism, reconstruction and recovery of HPLFs. The poor state of HPLFs under hypoxic conditions may therefore initiate or aggravate periodontal disease.

## Introduction

Periodontal disease is one of the most common infectious diseases and the primary cause of tooth loss. Currently, 14,000,000 individuals live on plateaus, where morbidity due to periodontal disease increases with altitude (1). A survey conducted by the Health Organization revealed that the morbidity of periodontal disease is 50% on the plains, whereas it increases to 70.4% on plateaus (2). One of the most important causes of the high morbidity is the low oxygen conditions on plateaus (3), as the air is thin and therefore oxygen content is low. Thus, the blood becomes dense, sticky and cohesive, leading to various degrees of hypoxia in body tissues (4). Changes in oxygen concentrations, an important physiological and pathological regulator, may affect the entire life span, from embryogenesis and development to the maintenance of normal function, dysfunction, disease and aging. Physiological oxygen tension (pO_2_) in normal tissues ranges from 24 to 66 mmHg (3–9% O_2_) (5) on plateaus, particularly at a higher altitude and ambient atmosphere. The oxygen that is utilized by metazoan gradually decreases, resulting in pathological hypoxia (pO_2_<3%). The periodontium is particularly sensitive to hypoxia, and a series of pathological and physiological reactions to hypoxia lead to the reduction of tissue defenses (6). Furthermore, hypoxia results in the decrease of redox potential, which leads to the gathering of anaerobes (7). The interaction of these factors accelerates the initiation and development of periodontal disease. Studies regarding plateau periodontal disease have mostly been epidemiological surveys of small sectors and samples (8,9). Few studies regarding the initiation, development and outcomes of the disease have been conducted, and even fewer studies have been conducted on the biological behaviors of periodontium cells under plateau circumstances, such as hypoxia or ultraviolet rays.

Of all the cells in the periodontium, human periodontal ligament fibroblasts (HPLFs) are the most numerous and have the most important role (10). They constantly produce new principal fibers and dental cement and reconstruct alveolar bones. HPLFs also synthesize the extracellular matrix, which not only holds the cells but also has special biological functions such as participating in cell conglutination, transportation and mineralization (10,11). If the number of HPLFs is reduced or the structure is broken, the periodontal support tissue may be damaged. This damage further induces or aggravates periodontal disease (12). Therefore, studies on the proliferation and mineralization activities of HPLFs are important in improving our understanding of the etiology and treatment of periodontal disease.

For the reasons stated previously, HPLFs were selected as the subject of this study. The effects of hypoxia on the proliferation, mineralization and ultrastructure of HPLFs at various time points were investigated by imitating different hypoxic conditions. To a certain extent, the results reveal the biological changes of HPLFs under hypoxic conditions. Therefore, this study provides an experimental basis for additional study on plateau-hypoxia-induced periodontal disease.

## Materials and methods

### Culture and identification of HPLFs

#### Sourcing and culturing of HPLFs

In this study, periodontal ligament tissues were isolated from the premolar teeth extracted from 6 donors (mean age: 13 years and 3 months) for orthodontic treatment. Informed consent was obtained from all of the patients prior to the beginning of experiments. Permission for a series of experiments was granted by the Ethics Committee of The Third Military Medical University (Chongqing, China). The patients were asked to gargle with chlorhexidine prior to the extraction of the tooth. Subsequently, the tooth was disinfected with 1% iodine and 72% alcohol. Following extraction, the tooth was repeatedly washed with sterile PBS and placed into a DMEM culture solution (Gibco, Carlsbad, CA, USA) containing 5% FBS. The tooth was subsequently sent to the super clean bench.

The tooth was placed in a sterile culture dish, and a small quantity of DMEM containing antibiotics (100 U/ml penicillin, 100 μg/ml streptomycin and 5 μg/ml amphotericin B; North China Pharmaceutical, Shijiazhuang, China) was added in order to keep the root face moist. The central third of the periodontal tissue on the root was scraped and cut into small sections (~1 mm^3^ each). The sections were spread evenly at the bottom of a 25 mm^2^ sterile culture bottle with DMEM infiltration. The bottom of the bottle was turned upward. Subsequently, 4 ml DMEM containing 5% FBS and antibiotics (Gibco) was carefully added to the bottle, which was subsequently placed into a 37°C CO_2_ incubator for 2–4 h. The bottom of the bottle was turned downward after the tissue sections had adhered in order to allow the culture solution to slowly cover the tissue sections. The solution was cultured again in the incubator and changed every four days. The growth condition of the cells was observed each day under an inverted microscope (Olympus, Tokyo, Japan). The cells were passaged at a ratio of 1:2 when they detached from the tissue sections and covered 80–90% of the bottom of the bottle.

#### Growth curve and doubling time of HPLFs

The HPLFs (2.0×10^4^/ml, fourth passage) were inoculated into a 24-well culture plate (Shanghai Jiang Lai Biotechnology Co., Ltd., Shanghai, China) at 1 ml for each well. Three wells were selected for cell counting every day. The growth curve was created following eight days of continuous observation.

The doubling time was T_D_ = t × log2 / (logN_t_-logN_0_), where t is the culture time, N_0_ is the number of cells at the beginning of the culture period, and N_t_ is the number of cells at the end of the culture period.

#### Identification of HPLFs using avidin-biotin complex (ABC) immunohistochemistry (IHC)

The HPLFs (1×l0^5^/ml, fourth passage) were inoculated into a culture dish with a glass slide in order to create a cell climbing slide. Paraformaldehyde was used for fixation and exclusive serum (bovine serum albumin 1 g, phosphate-buffered saline 100 ml, sodium azide 0.08 g) for closure. The first, second and third antibodies were added in sequence. DAB was primarily used in the color reaction, which was terminated by PBS. Subsequently, haematoxylin was used for the counterstaining process, and neutral balsam was used in the sealing process. Images were captured under a microscope (Olympus).

#### HPLF groups according to various hypoxic conditions

HPLFs were assigned into four groups, as follows: slight hypoxia group, 5% O_2_ content; middle hypoxia group, 2% O_2_ content; severe hypoxia group, 1% O_2_ content; and the control group, 21% O_2_ content. HPLFs in the three hypoxia groups were cultivated in a three-gas (CO_2_/O_2_/N_2_) incubator (NuAire Inc., Plymouth, MN, USA), and the HPLFs in the control group were cultivated in a common CO_2_ incubator (NuAire, Inc., Plymouth, MN, USA).

#### Effects of different hypoxic conditions on the proliferation of HPLFs at various time points

HPLFs (1.7×10^4^/ml, fifth passage) were inoculated in a 96-well culture plate at 200 μl/well. Each group contained four duplicate holes. The HPLFs in the different groups were cultivated according to their corresponding circumstances. Firstly, 20 μl of 3-(4,5-dimethylthiazol-2-yl)-2,5-diphenyltetrazolium bromide (MTT) solution (5 g/l) was added to each tested well 12, 24, 48 and 72 h after cultivation. Subsequently, cultivation was continued for a further 4 h at 37°C. The culture solution was discarded, and 20 μl DMSO was added to each tested well. The optical density (OD) of each tested well was measured using an ELISA plate reader (Precision Microplate Reader, Molecular Devices, Bio-Rad Inc., Hercules, CA, USA) at 490 nm following agitation for 10 min.

#### Effects of different hypoxic conditions on the ALP activity of HPLFs at various time points

HPLFs (3.7×10^4^/ml, fifth passage) were inoculated into a 96-well culture plate at 200 μl/well. Each group contained four duplicate holes. The HPLFs in the different groups were cultivated according to their corresponding circumstances. The culture solution was discarded 12, 24, 48 and 72 h after cultivation and the cells were washed with PBS.

Triton X-100 (Henan Sino-American Biotechnology Co., Ltd., Henan, China) was used to dissolve the cells. The lysates were maintained at 4°C overnight. The lysates from each well were moved to an Eppendorf tube (Eppendorf, Hamburg, Germany) for centrifugation at 997 × g for 5 min. Subsequently, 30 μl of supernatant fluid was extracted for the ALP activity detection using the ALP kit (R&D Systems, Minneapolis, MN, USA), according to the manufacturer’s instructions in the. An ultraviolet spectrophotometer (Unico, Franksville, WI, USA) was used to measure the OD at 520 nm.

#### Effects of severe hypoxia on the morphology of HPLFs at various time points

HPLFs (fifth passage) were assigned to the severe hypoxia and control groups, and the growth conditions were observed using an inverted microscope 12, 24, 48 and 72 h post-cultivation. Following trypsinization and centrifugation, the HPLFs were fixed using glutaraldehyde, dehydrated with acetone, and embedded using epoxy resin-618 (Chenguang Research Institute, Sichuan, China). The HPLFs were subsequently solidified in an oven (60°C) and sliced into 1 μm samples using an ultrasonic wave slicer. Subsequently, ultrathin 50–70 nm slices were created. HPLF ultrastructures were observed using a transmission electron microscope (TEM) (Olympus) after being coloured, washed and dried.

#### Statistical analysis

Data were expressed as the means ± SD. Mean values were compared by single factor analysis of variance (ANOVA) and a paired t-test using SPSS 13.0 statistical software (SPPS Inc., Chicago, IL, USA). P<0.05 was considered to indicate a statistically significant difference.

## Results

### Cultivation and identification of HPLFs

**Figure 1:**
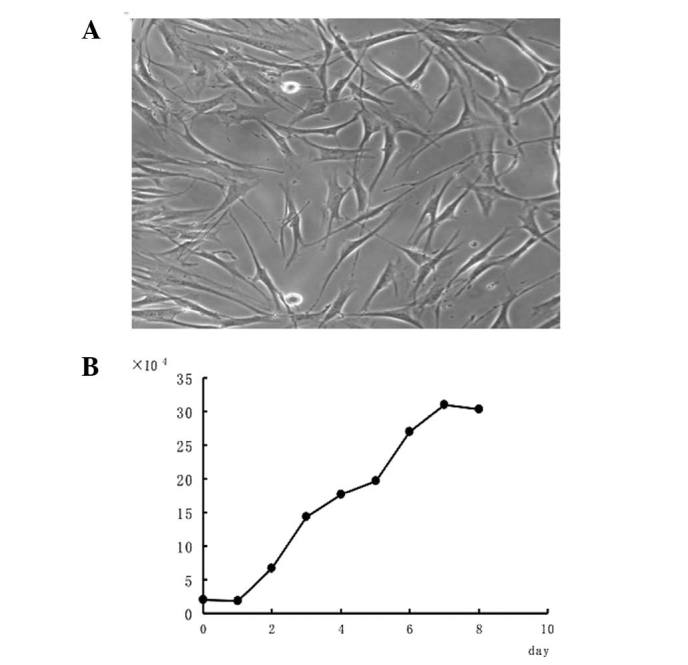
Cultivation of human periodontal ligament fibroblasts (HPLFs). (A) Fourth passage of HPLFs. Cells were either fusiform or dendroid in shape, with full cell bodies, clear nuclei, and two or three fine cytoplasmic processes. Original magnification, ×200. (B) Growth curve of HPLFs. The growth curve was similar to an upside down ‘S’ with the arrest, logarithmic growth, and plateau phases. The multiplication of HPLFs was completed within 35.6 h.

HPLFs began to migrate from the edge of the tissues following 48 h of cultivation. The coronal outgrowth appeared and extended after seven days, and the tissues disintegrated after two weeks. The HPLFs were fusiform in shape and in a good condition after being passaged. The cells were arranged in a sarciniform or swirl pattern (Fig. 1A). The HPLF growth curve was similar to an ‘S’, with arrest, logarithmic growth and plateau phases (Fig. 1B). HPLF multiplication was completed within 35.6 h.

**Figure 2:**
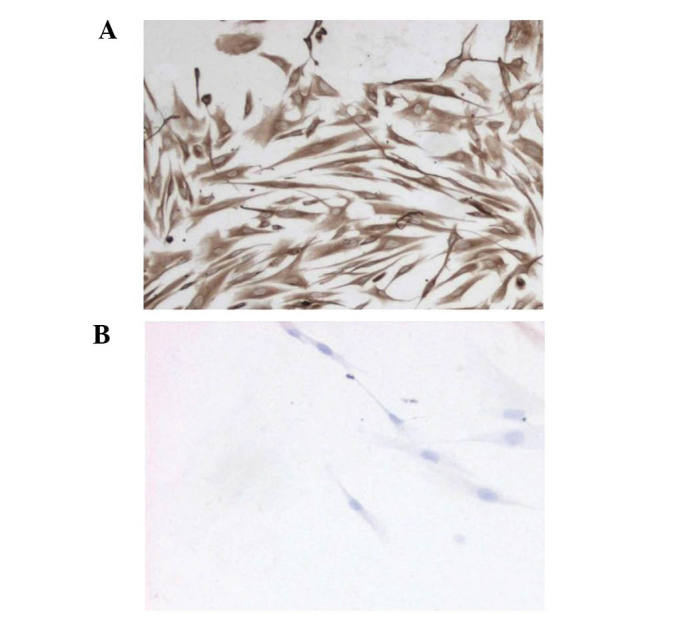
Identification of human periodontal ligament fibroblasts (HPLFs). (A) Vimentin expression in HPLFs. Vimentin was found in the cytoplasm with a yellow-brown color. Original magnification, ×200. (B) Keratin expression in HPLFs. Keratin was not found in HPLFs. The results proved that HPLFs are mesenchymal cells derived from the embryonic mesoderm. Original magnification, ×200.

IHC testing of the HPLFs (fourth passage) revealed that the cytoplasm was positive for vimentin, indicated with a yellow-brown color (Fig. 2A). However, keratin was not found in the cytoplasm (Fig. 2B). The results demonstrated that HPLFs are mesenchymal cells derived from the embryonic mesoderm.

### Effects of different hypoxic conditions on the proliferation of HPLFs at various time points

**Figure 3:**
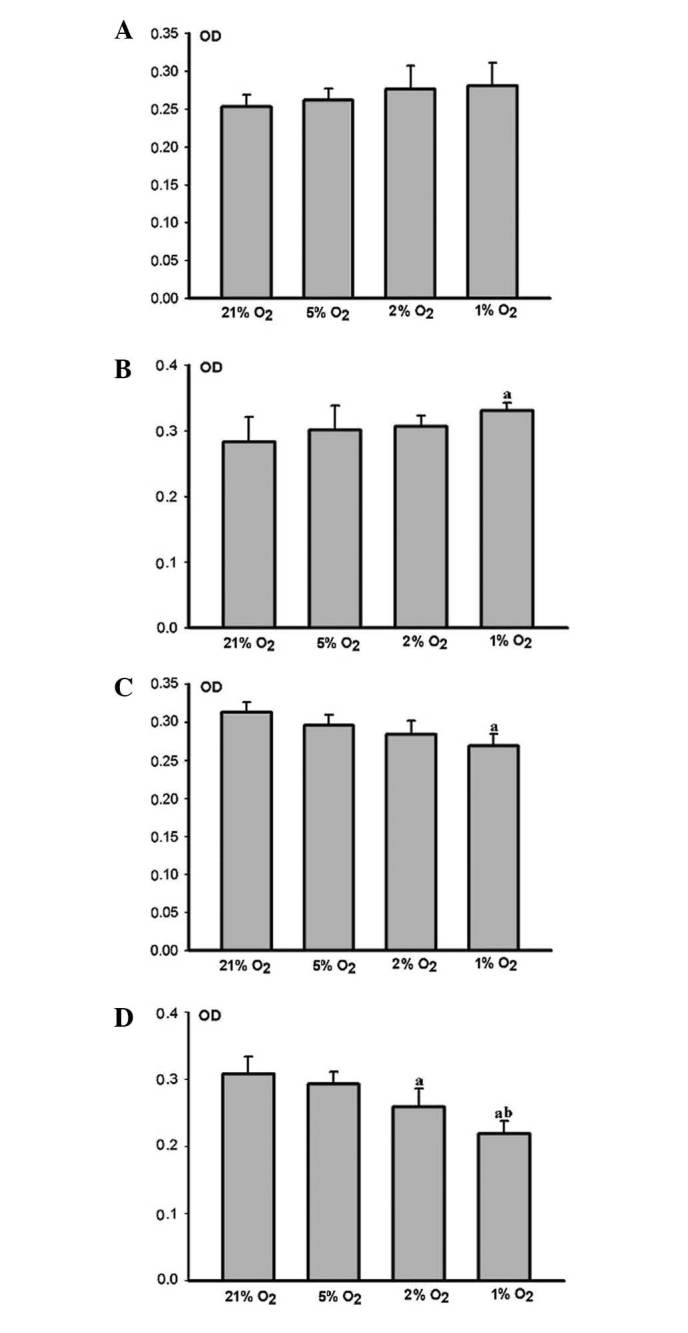
Effects of different hypoxic conditions on the proliferation of human periodontal ligament fibroblasts (HPLFs) at various times. n=4, ^a^vs. 21% O_2_ P<0.05, ^b^vs. 2% O_2_ P<0.05 (ANOVA and paired t-test). (A) At 12 h post-cultivation, the HPLFs grew more rapidly as the degree of hypoxia was increased, and there was no clear difference between the groups. (B) At 24 h post-cultivation. The HPLFs grew more rapidly as the degree of hypoxia was increased. Cell proliferation in the severe hypoxia (1% O_2_) group was significant. (C) At 48 h post-cultivation, the growth of HPLFs was restrained as the degree of hypoxia was increased. Cell proliferation in the severe hypoxia group was markedly restrained. (D) At 72 h post cultivation. HPLF growth was restrained as the degree of hypoxia was increased, cell proliferation in the middle (2% O_2_) and severe hypoxia groups was markedly restrained. The restraint was more visible in severe hypoxia.

The HPLFs grew more rapidly as the degree of hypoxia increased, when compared with the matched control group 12 h (Fig. 3A) and 24 h (Fig. 3B) post cultivation. Cell proliferation in the severe hypoxia group 24 h post-cultivation was considered to be significant (P<0.05) (Fig. 3B).

HPLF growth was restrained as the degree of hypoxia increased, when compared with the matched control group 48 h (Fig. 3C) and 72 h (Fig. 3D) post-cultivation. Cell proliferation in the middle and severe hypoxia groups 72 h post-cultivation was markedly restrained (P<0.05) (Fig. 3D). However, the restraint was more marked in the severe hypoxia group (P<0.05) (Fig. 3D).

### Effects of different hypoxic conditions on the ALP activity of HPLFs at various time points

**Figure 4:**
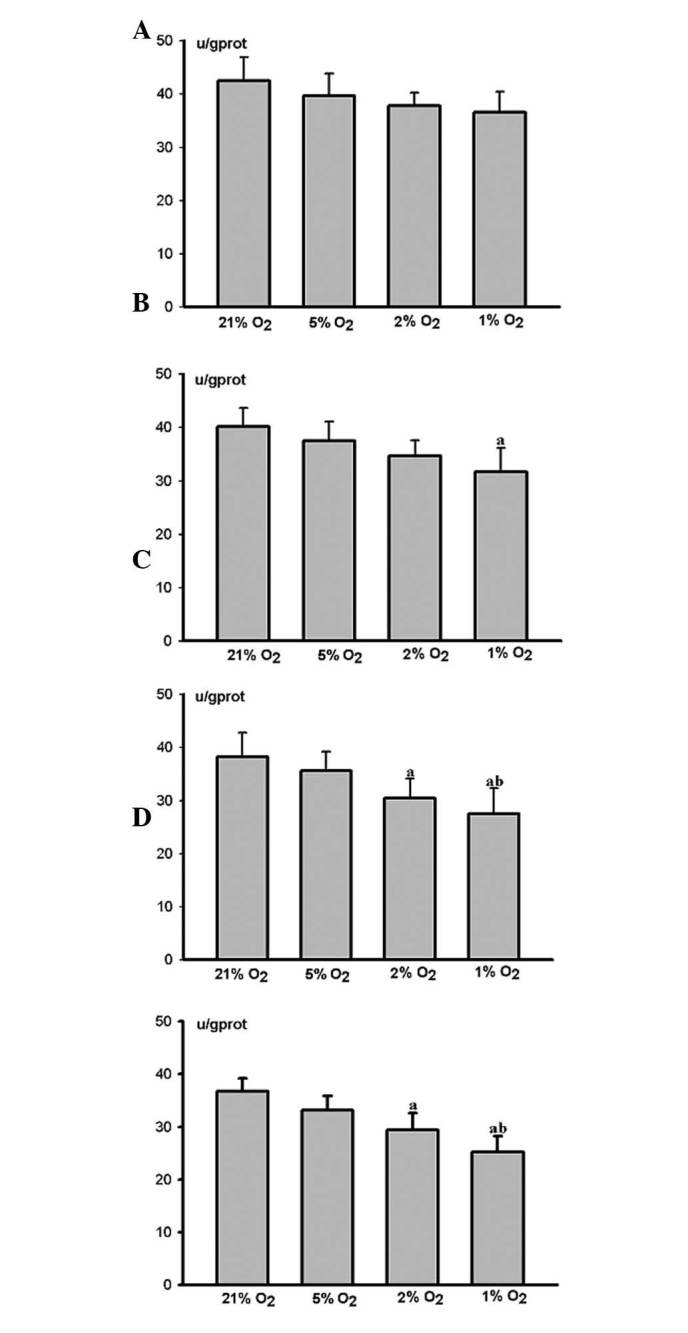
Effects of different hypoxic conditions on the alkaline phosphatase (ALP) activity of human periodontal ligament fibroblasts (HPLFs) at various time points: n=4, ^a^vs. 21% O_2_ P<0.05, ^b^vs. 2% O_2_ P<0.05 (ANOVA and paired t-test). ALP activity shows a decrease at each time point as the degree of hypoxia increases. (A) At 12 h post-cultivation, no clear difference was observed between the hypoxic and control groups. (B) At 24 h post-cultivation, the ALP activity of HPLFs in the severe hypoxia (1% O_2_) group was markedly restrained. (C and D) At 48 and 72 h post-cultivation, the ALP activities of HPLFs in the middle (2% O_2_) and severe hypoxia groups were markedly restrained. The restraint was clearest in the severe hypoxia group.

ALP activity decreased at each time point as the degree of hypoxia increased. No marked difference was observed between the hypoxic and control groups after 12 h (Fig. 4A). The ALP activity of the HPLFs in the severe hypoxia group was markedly restrained (P<0.05) after 24 h (Fig. 4B), whereas that of the HPLFs in the middle and severe hypoxia groups was restrained (P<0.05) after 48 h (Fig. 4C) and 72 h (Fig. 4D). However, the restraint was more marked in the severe hypoxia group (P<0.05) (Fig. 4C and D).

### Effects of severe hypoxia on the morphology of HPLFs at various time points

**Figure 5:**
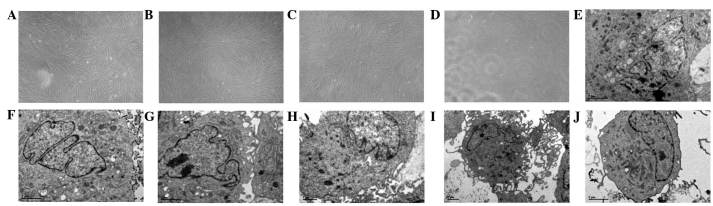
Effects of severe hypoxia on the morphology of human periodontal ligament fibroblasts (HPLFs). (A–D) Inverted microscopy was performed at various time points. (A) At 12 h post-cultivation the cells were in good condition, flat fusiform or dendroid in shape and were arranged in a monolayer with normal cell spacing; original magnification, ×100. (B) At 24 h post-cultivation, the cells grew vigorously with full cell bodies, clear nuclei and two or three fine cytoplasmic processes; original magnification, ×100. (C) At 48 h post-cultivation, the cells became contracted and sparse, their cytoplasmic processes were reduced and their cytoplasms became vesiculated; original magnification, ×100. (D) At 72 h post-cultivation, the cells were in a worse condition and sections disassembled and disappeared; original magnification, ×100. (E–J) Transmission electron microscope (TEM) was also performed at various time points. At 12 h post-cultivation revealed that the organelles remained normal in cells, and the cytoplasm of the HPLFs contained numerous mitochondria and rough endoplasmic reticula (RER); original magnification, ×6200. (F) At 24 h post-cultivation showed a marked increase in the number of mitochondria and RER; original magnification, ×6200. (G) At 48 h post-cultivation, the number of mitochondria and RER decreased. The mitochondria increased in size, the cristae appeared to be vague and RER structural disorder was observed; original magnification, ×6200. (H) At 72 h post-cultivation, degeneration occurred, and the number of mitochondria and RER further decreased with the broken membrane structure. Mitochondrial cristae were disassembled, vacuolar degeneration occurred and particles of the RER were reduced with increasing number of lysosomes; original magnification, ×6200. (I) At 24 h post-cultivation, the cells had numerous cytoplasmic processes; original magnification, ×5800. (J) At 72 h post-cultivation, the cell had few cytoplasmic processes; original magnification, ×6200.

Inverted microscopy at 12 h post-cultivation (Fig. 5A) revealed that the HPLFs adhered completely and were either fusiform or dendroid in shape and assembled as a monolayer. Following 24 h (Fig. 5B), the HPLFs grew vigorously, had full cell bodies, clear nuclei, and two or three fine cytoplasmic processes. Following 48 h (Fig. 5C) and 72 h (Fig. 5D), the HPLFs became contracted and sparse, and their cytoplasmic processes were reduced. Their cytoplasms were vesiculated, and a section of the HPLFs dismantled and disappeared.

TEM demonstrated at 12 h post-cultivation (Fig. 5E) that the HPLF cell organelles remained normal with clear nuclei and karyotheca. The cytoplasms of the HPLFs contained numerous rough endoplasmic reticula (RER) and mitochondria. Following 24 h (Fig. 5F), the number of mitochondria and RER significantly increased, and the mitochondria and RER exhibited mild expansions with complete membrane structures. The cell with a large or double nuclei exhibited more cytoplasmic processes (Fig. 5I). After 48 h (Fig. 5G), the number of mitochondria and RER decreased. The mitochondria increased in size, the cristae appeared vague, and the RER were structurally disordered. The number of cytoplasmic processes also decreased. Following 72 h (Fig. 5H), the HPLFs degenerated, and the number of mitochondria and RER decreased further with broken membrane structures. The mitochondrial cristae were broken, vacuolar degeneration occurred, RER particles reduced as the number of lysosomes increased and the number of cytoplasmic processes decreased further (Fig. 5J).

## Discussion

HPLFs were isolated and cultured according to the tissue culture method. IHC test results revealed that the cells were derived from the embryonic mesoderm. The HPLFs grew vigorously with full cell bodies and clear nuclei prior to the 10th passage. The HPLFs were fusiform in shape and in a good condition. The fourth to seventh passages were selected for this study as the cells in this period proliferated vigorously and had the best activity.

Cell proliferation is one of the most important factors in maintaining the balance of cell numbers and maintaining normal organism functions. We investigated the cell proliferation statuses under various hypoxic conditions at 12, 24, 48 and 72 h, respectively. In this study, O_2_ conditions ≤5% were termed hypoxic and 21% O_2_ conditions were termed control conditions. Cell viability was assessed following HPLF exposure to hypoxic conditions for various periods of time. Our results demonstrated that HPLF growth accelerated with an increase in the degree of hypoxia during acute hypoxia. However, growth was restrained as the degree of hypoxia increased over time. These findings are in accordance with several other studies (13–15). Harada *et al*(16) found that short-term hypoxia promoted the proliferation of fibroblasts in the heart. Lennon *et al*(17) found that the number of osteoblasts increased under short-term hypoxia. Ren *et al*(18) concluded that the number of bone marrow stromal cells markedly increased compared with the control group under short-term hypoxic conditions (8% oxygen content). However, the overall number of cells decreased over time. Piret *et al*(19) investigated whether hypoxia creates protective or destructive effects. The effects are directly related to the duration and degree of hypoxia. The effects of hypoxia on cell proliferation are determined using hypoxia inducible factor-1 (HIF-1) (20). HIF-1 is mostly composed of oxygen-sensitive (HIF-1α) and oxygen-insensitive subunits (HIF-1β) (20). HIF-1α may be degraded rapidly by hydroxyprolinase under normal conditions. However, hydroxylation is halted under hypoxic conditions (21). An excessive quantity of HIF-1α is capable of both promoting (short-term hypoxia) and restraining (extension of time) cell proliferation (21).

ALP is important for differentiating osteoblast-like cells, whose degree of activity reflects the mineralization ability of tissues and cells and the parameters for the formation of osteogenic property (22). This study revealed that ALP activity was decreased at each time point as the degree of hypoxia increased. The restraint was observed in the middle and severe hypoxia groups over time. These findings are consistent with those of earlier studies. Ren *et al*(18) stated that hypoxia was capable of restraining the mineralization ability of bone marrow stromal cells. Utting *et al*(23) demonstrated that the biological activities of osteoblasts *in vitro* were entirely oxygen dependent. Furthermore, hypoxia may markedly reduce the ALP activity of osteoblasts and the mRNA expressions of ALP and osteocalcin over time. The formation rate of bone-mineralized nodules was significantly reduced as the degree of hypoxia increased (24,25).

To demonstrate the effects of hypoxia on HPLFs, we used an inverted microscope and TEM to observe structural changes in HPLFs under severe hypoxic conditions. Furthermore, the effects of hypoxia on HPLFs were explored by observing the cell morphologies. It was observed under an inverted microscope that over time the HPLFs became smaller under severe hypoxic conditions. The growth and metabolism of HPLFs were suppressed, and the structures were broken (even necrotic). TEM revealed that the mitochondrial structures and RER of the HPLFs were broken as the time period under which the cells were exposed to severe hypoxic conditions was prolonged. Additionally, the number of cytoplasmic processes decreased while the number of lysosomes increased. The broken structure of the mitochondria directly affects the energy metabolism and protein synthesis (26). The changes in the RER revealed the slow rate of cell proliferation and division. The changes also revealed a dysfunction in protein synthesis (27). The reduced number of cytoplasmic processes demonstrated that the number of substances secreted and synthesized by cells were reduced. The increased number of lysosomes was a sign of cytotoxity. It revealed that large quantities of aging organoid and external harmful substances had gathered inside the cells (28). Therefore, cell proliferation and protein synthesis were restrained and an increased cytotoxicity occurred as the period of hypoxia was prolonged.

In conclusion, short-term and slight hypoxic conditions had relatively small effects on HPLFs, whereas long-term and middle or severe hypoxic conditions had negative effects on the proliferation and mineralization of HPLFs. Furthermore, the mitochondria and RER of HPLFs were broken under long-term severe hypoxic conditions. Therefore, middle or severe hypoxia in the long term is capable of affecting the reconstruction and recovery of periodontal tissues and may further initiate or aggravate periodontal disease.

## Abbreviations

ALP: proliferation and alkaline phosphatase
HPLFs: human periodontal ligament fibroblasts
RER: rough endoplasmic reticulum
